# Psychiatric disorders in older adults with cancer referred to psycho-oncology service in a tertiary care cancer centre: a 7-year real world experience from India

**DOI:** 10.1186/s12888-024-05492-x

**Published:** 2024-01-12

**Authors:** Jayita K. Deodhar, Lekhika N. Sonkusare, Savita S. Goswami

**Affiliations:** 1 Department of Palliative Medicine, Tata Memorial Hospital, Homi Bhabha National Institute, Dr. E. Borges Road, Parel, 400012 Mumbai, India; 2 Department of Psycho-oncology, Tata Memorial Hospital, Homi Bhabha National Institute, Dr. E. Borges Road, Parel, 400012 Mumbai, India

**Keywords:** Cancer, Geriatric psychiatry, Psycho-oncology, Psychiatric disorder, Developing country

## Abstract

**Background:**

Cancer affects mental health in older adults with cancer (OAC), affecting almost 50% of the patients. There are only a few studies on psychiatric disorders in OAC, especially in low resource settings. We report on our real-world experience of prevalence of and factors associated with psychiatric disorders in OAC referred to a psycho-oncology service in an Indian tertiary care cancer institute.

**Methods:**

We retrospectively analysed medical and psycho-oncology records of patients aged 60 + on cancer-directed treatment or follow-up for < 2 years after treatment completion, referred to psycho-oncology services in a tertiary care cancer centre in Mumbai, India, from Jan 2011-Dec 2017. We recorded sociodemographic, clinical, and treatment-related variables, as well as past psychiatric disorders. The ICD-10 was used to record current psychiatric disorder type and presence. IBM SPSS version 24 (Armonk, NY, USA) was used for descriptive measures, tests of association, and logistic regression analysis. The study protocol was approved by Institutional Ethics Committee and registered with the Clinical Trials Registry-India (CTRI/2020/06/026095).

**Results:**

Of 763 patients included in the study, 475 (62.3%) were males and 436 (57.1%) were inpatients, with a median age of 65 years. 93% of the patients had a solid tumour and 207 (27.1%) had a history of psychiatric disorder. A current psychiatric diagnosis was noted in 556 patients (72.9%) on initial presentation, of which adjustment disorders, delirium and depression and anxiety disorders were most frequently seen in 25.2%, 21% and 11.1%, respectively. On univariate analysis, a past history of psychiatric disorders (χ2 = 34.6, *p* < 0.001), lower performance status (χ2 = 9.9, *p* = 0.002) and haematolymphoid malignancy (χ2 = 4.08, *p* = 0.04) significantly increased the risk of current psychiatric diagnosis. Logistic regression confirmed these variables as significant.

**Conclusion:**

Older adults with cancer referred to psycho-oncology services have high rates of psychiatric disorders at their initial presentation, mainly adjustment disorders, delirium and depression and anxiety. A past history of psychiatric disorders, lower performance status and haematolymphoid cancers significantly increased the risk of psychiatric disorders. Multidisciplinary psycho-oncology teams including a psychiatrist should be integrated in comprehensive care of this group of patients. Further research outcomes and effect of psycho-oncological interventions is required in older adults with cancer in LMIC settings.

## Introduction

Cancer in the older adults (OA) is an increasing public health burden, accounting for 50% of cancer cases in developed nations [1]. Cancer affects their mental health, with about 44% of the older adult cancer patients experiencing psychological distress [2].

Several authors have reported that psychiatric disorders are prevalent in almost 50% of OA with cancer [3–5]. Psychiatric disorders commonly seen in this population are depression, anxiety and delirium ( [3, 6]. Authors have reported that 40% of geriatric cancer patients have anxiety symptoms/disorders [7]. Lower socio-economic and educational, and single status were associated with both higher distress and higher prevalence of psychiatric disorders ( [3]. Depression is common in those with advanced stages, severe pain and poor general condition [6]. A few studies have addressed the problem of psychiatric disorders in geriatric cancer patients in low- and middle-income countries [8].

India’s geriatric population is steadily increasing. It is estimated that by 2026, around 450,000 men and 370,000 women aged 60 years and above will have cancer [9]. Authors of a systematic review reported that psychiatric disorders like depression are present in almost 43–80% of older adults attending general hospital clinics in India [10].

Most of the studies on mental health issues in older adults with cancer have been done in high income countries, in oncology specific settings or geriatric oncology clinics, using different rating scales for screening or identification. No studies from India have reported on prevalence of or factors associated with psychiatric disorders in older adults with cancer in the setting of a psycho-oncology service.

The psycho-oncology service in our tertiary cancer care hospital includes psychologists and psychiatrists, runs on a consultation-liaison model and cares for patients in all age groups, at any point of disease trajectory. The annual number of patient assessments is approximately 3000–3300 patients. For this study, we aimed to evaluate the rates and types of psychiatric and associated factors in older adult patients with cancer referred to our psycho-oncology service in a tertiary cancer centre in India.

## Methodology

We did a retrospective analysis of medical and psycho-oncology records of routinely collected data of all patients 60 years and above, newly referred to psycho-oncology service in a tertiary care cancer centre in Mumbai, India from January 2011 to December 2017. For this study, we included outpatients and inpatients on active cancer-directed treatment, including supportive care, or attending follow-up for less than two years after treatment completion. Patients on best supportive care alone (those not on any cancer-directed treatment) were excluded. Incomplete patient records were excluded. Patients were evaluated at initial referral by clinical assessment interviews using standard psycho-oncology and psychiatry assessment forms, developed by the service for routine practice. The performance status of the patients -a measure of cancer patients’ symptoms and functioning depending on their ambulation and care need- was marked using the Eastern Cooperative Oncology Group Scale, higher grades indicating worse status [11]. Pain and fatigue were evaluated as standard assessment by using numerical rating scale (NRS) ranging from 0 to 10 [12, 13]. A diagnosis of psychiatric disorder (a set of clinical symptoms or behaviors causing subjective distress and socio-occupational functioning impairment) was made according to the International Classification of Diseases-10th edition [14].

## Data collection

We collected data from the hospital electronic medical records and the departmental psycho-oncology and psychiatry assessment forms. Patients’ demographic data included age, sex, marital and employment status, inpatient or outpatient status and paying category for hospital charges (general or private). Clinical variables recorded were cancer diagnosis, treatment received and comorbid conditions. We noted family and past history of psychiatric disorders and psychiatric treatment. Pain and fatigue scores on NRS were noted from the medical charts. The presence and type of current psychiatric disorder according to ICD-10 were recorded.

The outcomes were the number of patients diagnosed with a psychiatric disorder at initial psycho-oncology assessment, the types of and factors associated with presence of current psychiatric disorders.

### Statistical analysis

Descriptive measures of frequency and percentages, means with standard deviation and median with interquartile range were used for continuous variables. Chi square tests were used to examine association between psychiatric disorders and demographic and clinical variables. Those factors which were significant predictors of the psychiatric disorders (*p* < 0.05) on univariate analysis were included in the multivariate model using binary logistic regression. All P values were done using a two-sided hypothesis with CIs at the 95% level. *P* < 0.05 was considered significant. Analysis was done using IBM SPSS version 24 (Armonk, NY).

The study protocol was approved by Institutional Ethics Committee and registered with the Clinical Trials Registry-India (CTRI/2020/06/026095).

## Results

### Participant characteristics

In the study period, 763 new patients of 60 years and above were referred to the psycho-oncology service, of whom 475 (62.3%) were males and 436 (57.1%) were inpatients. The mean age was 66.1 years (+/- 5.5) and median age was 65 years. Just above 50% of the patients could pay the standard hospital care charges. The most common cancer diagnoses were head and neck, (187, 24.5%), lung (84, 11.1%) and breast (83,10.9%) (Table 1).

**Table 1:** Patient characteristics (*N* = 763)

Variable	n	%
**Sex**		
Male	475	62.3
Female	288	37.3
**Age**		
60–74 years	698	91.5
75 years and above	65	8.5
Median age Mean age (SD)	65 66.1 (5.5)	
**Admission status**		
Inpatients	436	57.1
Outpatients	327	42.9
**Cancer sites**		
Head &Neck	187	24.5
Lung	84	11.1
Breast	83	10.9
Esophagus and stomach	67	8.8
Gynecological	63	8.2
Urological	58	7.6
Gastrointestinal	56	7.3
Hepaticopancreaticobiliary	51	6.7
Hematolymphoid	48	6.3
Brain tumours	39	5.1
Bone and soft tissue	27	3.5
**Treatment intent**		
Curative	656	85.9
Palliative	101	13.2
Follow up	6	0.9
**Performance status**		
ECOG 0–2	564	73.4
ECOG 3–4	195	25.6
(Missing 4)		
**Co-morbidities**		
No	364	47.7
Yes	359	47
Missing data	41	5.3
**Past history of psychiatric disorder**		
Absent	556	72.9
Present	207	27.1

At presentation to Psycho-oncology service, most of the patients were on curative intent cancer-directed treatment (656, 85.9%). Almost 75% of the patients had a performance status of 0–2 on the European Cooperative Oncology Group (ECOG) assessment scale. Comorbidities were present in 359 (47%) patients. More than a quarter of the patients (207, 27.1%) patients had a past history of psychiatric disorder (Table 1).

### Psychiatric disorders

Five hundred and fifty-six (72.9%) patients were diagnosed with psychiatric disorder, on their initial assessment in psycho-oncology service according to ICD-10 classification system. The most common psychiatric disorders were adjustment disorders (192, 25.2%), delirium (160, 21%) and depressive and anxiety disorders (85, 11.1%) (Table 2).

**Table 2:** Psychiatric disorders in older adults with cancer (*N* = 763)

Psychiatric diagnosis	n	%
Yes	556	72.9
No	207	27.1
**Types of psychiatric disorder**		
Adjustment disorder	192	25.2
Depressive and anxiety disorder	85	11.1
Delirium	160	21
Psychotic and bipolar affective disorder	83	10.9
Substance use disorder	36	4.7

On univariate analysis, past history of a pre-existing psychiatric disorder (χ2 = 34.68, *p* < 0.001) lower performance status (χ2 = 9.9, *p* = 0.002) and hematolymphoid malignancies (χ2 = 4.08, *p* = 0.04) significantly increased the risk of psychiatric disorders in geriatric cancer patients referred to psycho-oncology service (Table 3). On multiple logistic regression, these factors remained significant, with a past history of psychiatric disorder being the most substantial (Odds Ratio 4. 66, CI 2.9–7.5), lower performance status (Odds Ratio 2.32, CI 1.5, 3.5)) and hematolymphoid malignancies (Odds Ratio 2.56, CI 1.1, 5.9) (Table 4). Age, sex, payment ability (regular/subsidized hospital charges), comorbidities and pain and fatigue levels were not significantly associated with presence of psychiatric disorders.

**Table 3:** Factors associated with psychiatric disorders in older adults with cancer

Variable	Psychiatric disorder absent (n, %)	Psychiatric disorder present (n, %)	Total	χ2	p-value
**Gender**					
Male	130 (27.4)	345 (72.6)	475	0.036	0.85
Female	77 (26.8)	211 (73.2)	288		
**Age**					
60-74y	191 (27.4)	507 (72.6)	698	0.227	0.63
75y and above	16 (24.7)	49 (75.3)	65		
**Ability to pay hospital charges**					
General category	95 (28.5)	238 (71.4)	333	0.585	0.44
Private category	112 (26.1)	318 (73.9)	430		
**Admission status**					
Outpatient	87 (26.6)	239 (73.3)	326	0.066	0.8
Inpatient	120 (27.5)	316 (72.4)	436		
**Cancer type**					
Solid	200 (27.9)	515 (72.1)	715	4.079	**0.04**
Haematolymphoid	7 (14.5)	41 (85.5)	48		
Performance status*					
ECOG 0–2	170 (30.2)	394 (69.8)	564	9.997	**0.002**
ECOG 3–4	36 (18.4)	159 (81.6)	195		
Pain severity*					
No-mild	118 (27.7)	307 (72.3)	425	1.252	0.26
Mod-severe	82 (31.7)	176 (68.3)	258		
Fatigue severity*					
No-mild	67 (29.3)	161(70.7)	228	0.002	0.96
Mod-severe	132 (29.2)	320 (70.8)	452		
**Past history of psychiatric disorder**					
Absent	183 (32.9)	373 (67.1)	556	34.68	**< 0.001**
Present	24 (11.5)	183 (88.4)	207		

*Missing data

Test used—Chi^2^ test

**Table 4:** Predictors of psychiatric disorders in older adults with cancer

Variables	n	Psychiatric disorder present	Psychiatric disorder absent	Odds Ratio (CI)	P value
Past history of psychiatric disorder					
No	556	373 (67.1%)	183 (32.9%)	4.66 (2.9, 7.5)	<0.001
Yes	207	183 (88.4%)	24 (11.5%)		
**Performance status**					
ECOG 0-2	564	394 (69.8%)	170 (30.2%)	2.32 (1.5, 3.5)	<0.001
ECOG 3-4	195	159 (81.6%)	36 (18.4%)		
**Type of cancer**					
Solid	715	515 (72.1%)	200 (27.9%)	2.56 (1.1, 5.9)	0.027
Haemato-		41 (85.5%)	7 (14.5)		
lymphoid	48				

Test used—Binary logistic regression

## Discussion

Our study is the first large report with real world data of clinically diagnosed psychiatric disorders in older adults with cancer (OAC) at their initial presentation to psycho-oncology service in India. We found that 73% of OAC had a clinically diagnosed psychiatric disorder. A past history of psychiatric disorder, a lower performance status and a diagnosis of hematolymphoid malignancy significantly increased the risk of a psychiatric diagnosis at presentation to psycho-oncology.

Almost 75% of older adults with cancer referred to psycho-oncology service had a psychiatric diagnosis, which is higher than the rates of 20–43% reported in other studies [2, 15]. We conducted our study in the psycho-oncology service and included patients with any cancer site. Identification of distress (an unpleasant subjective psychological, social and spiritual experience resulting from multiple factors and affecting coping ability in patients with cancer) at the initial screening in the oncology outpatient clinic would have triggered the referrals to our psycho-oncology service, resulting in a high percentage of psychiatric disorders in the referred patients we assessed [16]. The other studies used rating scales, focused on a single cancer site and did not diagnose according to a structured classification system.

We found that adjustment disorders, depression, and anxiety accounted for 36% of psychiatric disorders, as diagnosed on clinical interviews. Other studies have found similar rates of depression or anxiety symptoms but these used rating scales in older adults with a single cancer or on chemotherapy [17, 18]. Agustini found that depressive symptoms were significantly associated with cancer [19].

Delirium is a neuropsychiatric syndrome, characterized by impairment in consciousness, arousal, attention, emotions, psychomotor behaviors and other aspects of cognition, including memory and thinking, which can be precipitated by physical illnesses, infection, etc. [14, 20].. Increasing age is an important predisposing factor. We found that 21% of the study participants were diagnosed with delirium, which is significant clinically as delirium needs to be identified for prompt management to reduce morbidity and mortality. Only one previous study has reported cognitive impairment in older adults receiving chemotherapeutic treatment [18]. We included inpatients in our study which could explain the high rates of delirium in this group of OAC.

Interestingly, 10% of the study patients had a diagnosis of severe mental illness and 5% had a substance use disorder, which has hitherto not been documented in previous studies in this group of patients. Older patients with history of psychotic or bipolar affective disorder who are diagnosed with cancer need comprehensive care for managing the psychiatric disorder along with the cancer treatments, with a particular focus on symptom assessment and medication interactions.

A past history of psychiatric disorder and poor performance status increased the risk of psychiatric disorders in the OAC in our study, like the findings in Lee et al.’s systematic review [3]. We found, interestingly, that OACs with a hematolymphoid malignancy had a two times higher risk of psychiatric disorders, as compared to those with solid tumours. Studies have looked at psychiatric disorders in older adults with solid and hematolymphoid malignancies separately, but there have been no head-to-head comparisons between these two cancer groups [21]. In our study, the patients with hematolymphoid malignancies had more of adjustment disorders and delirium as compared to those with solid tumours. The possible reasons for these psychiatric disorders in older patients with hematolymphoid malignancies could be stress due to intensive chemotherapy schedules, treatment-related side-effects, and infection.

Gender, physical symptoms like pain and fatigue, and number of comorbidities did not significantly increase the risk of current psychiatric disorders in our older adult patients with cancer. We did not find any association between paying ability (regular or subsidized) charges and a current psychiatric disorder. A possible explanation is that these factors were stressful for all patients undergoing treatment in our low resource setting.

Our study had a few limitations. We did a retrospective analysis and included patients referred to psycho-oncology service, resulting in referral bias. However, our study was a real-world experience, over a seven-year period and gave an overview of psychiatric disorders in the referred patients. Our patients included those who had a past history of psychiatric diagnosis (27% of the patients), which could account for the high rates of psychiatric disorders at initial assessment (73%) in psycho-oncology clinic. However, the overall rate for psychiatric disorders was much higher at 73%, which imply that other factors were more contributory. We also did not explore whether the patients who had a past history of psychiatric disorder were compliant with their psychotropic medications, if prescribed earlier. Interventions for management of the psychiatric disorders was not the focus for this current study. Also, we did not evaluate if patients had used psychotherapy and counselling service prior to their referral. We, therefore, could not evaluate if these factors were protective. We did not look at differences between patients who were on active treatment and those who were on follow up. Our study was in a single- centre in an urban tertiary cancer hospital, limiting the generalizability to other settings. We did not evaluate cancer symptoms in the psycho-oncology clinic as patients’ cancer-related symptom assessment and management were done in the oncology clinics. We did assess physical symptoms of pain and fatigue which are encountered commonly in psycho-oncology clinics and might affect the patients’ psychological status [22]. Our focus was on examining the presence of psychiatric disorders in the older adults referred to our psycho-oncology service. Hence, we did not do a differential analysis of cancer symptoms with the type of cancer diagnosed and with the time of cancer diagnosis.

The strengths of our study are that the study period covered 7 years, and included more than 700 older adults with cancer, referred to our psycho-oncology clinic. We used a clinical interview using ICD-10 for psychiatric diagnostic classification and not rating scales. We included patients with different site-specific cancers, including both solid and hematolymphoid.

Our findings of high rates of psychiatric disorders in older adults with cancer suggest that psycho-oncology services should be integrated in comprehensive care of this group of patients. Also, our study highlights the importance of including a psychiatrist in the psycho-oncology team, as almost 75% of the older adults with cancer had a psychiatric disorder according to standard diagnostic criteria, including delirium in 21% and severe mental illness in 10% of the patients. Management of older adults with cancer requires prompt and prolonged psychiatric evaluation, appropriate interventions and follow up.

## Conclusion

Almost 75% older Indian adults with cancer newly referred to psycho-oncology service had a psychiatric disorder at initial presentation. Adjustment disorders, delirium and depression and anxiety were the most frequent diagnoses. A past history of psychiatric disorders, lower performance status and hematolymphoid cancers were significantly associated with psychiatric disorders. These findings have implications for clinical service delivery in that psycho-oncology services need to be integrated in comprehensive care of older adults diagnosed with cancer and that the service should include a psychiatrist. Future research should include feasibility of comprehensive geriatric assessment in a psycho-oncology setting, use and effect of psychological and psychopharmacological interventions and outcomes of older adults with cancer with psychiatric disorders in LMIC settings.

## Data Availability

The datasets used and analysed during the current study are available from the corresponding author on reasonable request.
